# A Modeling Study of the Emergence of Eye Position Gain Fields Modulating the Responses of Visual Neurons in the Brain

**DOI:** 10.3389/fncir.2020.00030

**Published:** 2020-05-26

**Authors:** Daniel M. Navarro, Hannah E. Smithson, Simon M. Stringer

**Affiliations:** ^1^Oxford Centre for Theoretical Neuroscience and Artificial Intelligence, Department of Experimental Psychology, University of Oxford, Oxford, United Kingdom; ^2^Oxford Perception Laboratory, Department of Experimental Psychology, University of Oxford, Oxford, United Kingdom

**Keywords:** eye-position, gain modulation, visual cortex, neural network, self-organizing

## Abstract

The responses of many cortical neurons to visual stimuli are modulated by the position of the eye. This form of gain modulation by eye position does not change the retinotopic selectivity of the responses, but only changes the amplitude of the responses. Particularly in the case of cortical responses, this form of eye position gain modulation has been observed to be multiplicative. Multiplicative gain modulated responses are crucial to encode information that is relevant to high-level visual functions, such as stable spatial awareness, eye movement planning, visual-motor behaviors, and coordinate transformation. Here we first present a hardwired model of different functional forms of gain modulation, including peaked and monotonic modulation by eye position. We use a biologically realistic Gaussian function to model the influence of the position of the eye on the internal activation of visual neurons. Next we show how different functional forms of gain modulation by eye position may develop in a self-organizing neural network model of visual neurons. A further contribution of our work is the investigation of the influence of the width of the eye position tuning curve on the development of a variety of forms of eye position gain modulation. Our simulation results show how the width of the eye position tuning curve affects the development of different forms of gain modulation of visual responses by the position of the eye.

## 1. Introduction

Visual neuronal responses are often characterized in terms of the selectivity of the responses to the location of a given visual stimulus. The portion of the visual space in which a visual stimulus changes the responses of a neuron is conventionally referred to as the *receptive field*. For instance, the receptive field of neurons in the primary visual cortex (V1) is fixed to a visual space defined in a retinocentric frame of reference (Guo and Li, 1997; Morris and Krekelberg, 2019), whilst the receptive field of neurons within other areas of the primate dorsal visual pathway is fixed to a visual space defined in different body-centered reference frames such as head (Galletti et al., 1993; O'dhaniel et al., 2005) or hand (Buneo and Andersen, 2006; Bremner and Andersen, 2012), which are relevant for guiding motor actions. Visually responsive neurons in the cortex have been observed to fire maximally when visual stimuli are positioned in a particular *preferred location* within the receptive field. A key experimental observation that we investigate in the models presented below is that such visual responses are commonly reported to be modulated by bodily state or posture, e.g., position of the eyes, head, or hand (Andersen et al., 1985; Brotchie et al., 1995; Buneo et al., 2002; Pesaran et al., 2006; Bremner and Andersen, 2012). This *gain modulation* effect, however, does not influence the preferred location or the general response profile of visual neurons, but only the amplitude of the responses. In particular, different forms of eye position gain modulation have been observed in many cortical areas (Andersen and Mountcastle, 1983; Galletti and Battaglini, 1989; Lal and Friedlander, 1990; Brotchie et al., 1995; Galletti et al., 1995; Hoffmann, 1998; Trotter and Celebrini, 1999; Breveglieri et al., 2009; Merriam et al., 2013). For example, gain modulation by eye position in parietal neurons has been described as a multiplicative modulation of the underlying Gaussian retinotopic receptive field by a linear or saturating function of the position of the eye (Andersen and Mountcastle, 1983; Brotchie et al., 1995). Previous modeling studies investigating the development of multiplicative gain modulation used a linear function to model the response profiles of eye position input neurons (Salinas and Abbott, 1996). Linear functions, however, do not accurately represent the Gaussian response profile of eye position neurons in the cortex (Marĉelja, 1980; Andersen et al., 1987; Dayan and Abbott, 2001; Wang et al., 2007). Here we show how a variety of different functional forms of eye position gain modulation of retinotopic visual responses, including peaked and monotonic modulation, may develop with a more biologically realistic Gaussian function used to model the responses of eye position input neurons. Understanding how gain modulation may develop in the brain provides an insight into the way the brain encodes information that is relevant to high-level visual functions, such as coordinate transformation, eye movement planning, visual-motor behaviors, and stability of spatial awareness.

### 1.1. Physiology

The dependence of cortical visual responses on both the retinal location of visual stimuli and on the position of the eye (i.e., gaze direction) was first reported by Andersen and Mountcastle (1983). The following two independent tasks were used by the authors to investigate the responses of light-sensitive neurons in area 7a of the primate brain. In the first task a peripheral stimulus was flashed in a particular head-centered location whilst the monkey was fixating at some gaze angle. In the second task a stimulus was flashed on different screen locations and no restrictions were imposed over the freedom of eye movements. There were no head movements during either of the tasks. The authors reported that both tasks revealed an influence of the position of the eye on the responses of parietal visual neurons. For instance, the proportion of recorded neurons that had responses significantly changed by the position of the eye was 61% for the first task and 10% for the second task. Particularly during the first task neuronal responses were found to become three times stronger when the eye moved 20° toward the preferred eye position. These results led the authors to investigate how neuronal responses depended on the precise relationship between the retinotopic location of a visual stimulus and the position of the eye (Andersen et al., 1985). The later work of Andersen et al. (1990) reported these same effects in the lateral intraparietal area (LIP). Such gain modulated neuronal responses were characterized in Andersen et al. (1990) as a multiplicative interaction between a *monotonic* eye position modulation component and the Gaussian retinotopic receptive field.

Different forms of eye position gain modulation have also been observed in several brain areas (Galletti and Battaglini, 1989; Lal and Friedlander, 1990; Galletti et al., 1995; Hoffmann, 1998; Trotter and Celebrini, 1999; Breveglieri et al., 2009; Merriam et al., 2013). The work of Galletti et al. (1995) and Breveglieri et al. (2009) reported the presence of more *peaked* gain modulation by eye position in the primate parietal occipital area (PO). The experimental task in Breveglieri et al. (2009) showed a different quantity of retinotopic neurons in area V6A had either *peaked* or *monotonic* eye position gain modulation. The task consisted of presenting visual stimuli at fixation points in each of nine equally spaced fixation locations organized as a 3 × 3 grid. The authors reported eye position gain modulated responses in over 55% of recorded neurons. Moreover, the gain modulation of approximately 73% of these neurons was peaked, whilst only the remaining 27% were monotonic. The study of Merriam et al. (2013) showed the presence of neuronal responses in the human visual cortex modulated by the position of the eye. The task in Merriam et al. (2013) consisted of periodically rotating a visual stimulus around a fixation point. The same set of stimuli was presented for several different eye positions. Functional magnetic resonance imaging (fMRI) was used to measure cortical activity. The authors observed that eye position changed the amplitude of visual responses without affecting the retinal preference of the responses. All of these experimental studies (Galletti and Battaglini, 1989; Lal and Friedlander, 1990; Galletti et al., 1995; Hoffmann, 1998; Trotter and Celebrini, 1999; Breveglieri et al., 2009; Merriam et al., 2013) found that cortical responses to the retinal location of visual stimuli are multiplicatively modulated by the position of the eye.

### 1.2. Modeling Approaches

Previous modeling studies have attempted to explain how gain modulation may develop in the brain. The recent simulation study by De Meyer and Spratling (2011) showed that gain modulation can appear in a predictive coding model using unsupervised learning. In the context used by the authors, predictive coding is defined as neural theory based on the principle of minimizing error in the stimulus-driven activity between bottom-up and top-down predictions of the internal representation of the stimulus. Although predictive coding has been suggested to be implemented by cortical feedback connections calculating residual errors that would be then propagated by cortical feedforward connections, currently there is no physiological evidence of predictive coding being implemented on the neuronal level in the cortex (Bastos et al., 2012; Kwisthout and Van Rooij, 2013; M and S, 2015). In contrast, the influential work of Salinas and Abbott (1996) investigated the development of gain modulated responses using recurrent connections within a network model of cortical neurons. The recurrent connectivity was defined according to the distance between the preferred retinal locations of the neuronal responses. Although the model was based on ordinary properties of cortical circuits, the authors used a linear function to model the response profiles of eye position input neurons. This is an important issue in terms of biological plausibility because, although a variety of responses profiles have been observed in the brain, the response profiles of many eye position neurons have been reported to be *Gaussian* (Marĉelja, 1980; Andersen et al., 1987; Dayan and Abbott, 2001; Wang et al., 2007). Furthermore, the recurrent connections in their model violated the widely accepted Dale's law, which establishes that a presynaptic neuron cannot make both excitatory and inhibitory synaptic connections on postsynaptic neurons (O'Donohue et al., 1985). In addition, the model of Salinas and Abbott (1996) was not trained to *learn* visual responses that were multiplicatively modulated by eye position. Instead, the synaptic connections were hardwired to ensure that the visual responses of each neuron within the network were gain modulated by eye position. In this article we address the biological issues in Salinas and Abbott (1996) with both a hardwired neural network model and a self-organizing neural network model of the development of retinotopic visual responses modulated by eye position.

### 1.3. Our Work

In this work we investigate how a variety of different functional forms of gain modulation, including multiplicative gain modulation, may develop when a Gaussian function is used to model the responses of both eye position input neurons and retinocentric input neurons. Moreover, we investigate how the width of the Gaussian response profile of the eye position input neurons affects the linearity of the gain modulation by eye position of the retinotopic visual output neurons. In the first part of this article we study a hardwired model similar to Salinas and Abbott (1996). The visual input to our hardwired model consists of the sum of a term encoding the retinal location of the visual stimulus and a term encoding the position of the eye (i.e., the gaze direction). In contrast to Salinas and Abbott (1996), our model does not violate the Dale's Law (O'Donohue et al., 1985) and response profiles of the eye position input neurons and retinotopic visual input neurons were both modeled by a more biologically realistic (Gaussian) function. In the second part of this article we study a self-organizing model of the development of multiplicative gain modulation by eye position. Our simulation results again show the emergence of a variety of different functional forms of gain modulation. Moreover, the linearity of the gain modulation by eye position of the retinotopic visual output neurons is affected by the width of the Gaussian response profile of the eye position input neurons.

## 2. Hardwired Model

In this section we present simulation results investigating the development of multiplicative gain modulation by eye position. In particular, we address the following issues of a similar model by Salinas and Abbott (1996). First we use Gaussian functions to model both retinal stimulus position and eye position input components of each visual output neuron. In Salinas and Abbott (1996), the authors used a linear function to model the influence of the position of the eyes on the visual responses. Gaussian functions, however, have been reported to be a better fit to the response profile of parietal eye position neurons (Marĉelja, 1980; Andersen et al., 1987; Dayan and Abbott, 2001; Wang et al., 2007). Secondly, we show that multiplicative gain modulation can be obtained without violating the Dale's Law (O'Donohue et al., 1985). Finally, the firing rate responses were modeled with a sigmoid transfer function rather than the linear function used by (Salinas and Abbott, 1996). A sigmoid transfer function represents more accurately the firing rate profile of individual neurons observed in the cortex (Marĉelja, 1980; Dayan and Abbott, 2001). Furthermore, we show how the width of the eye position tuning curve affects the overall linearity of gain modulation by eye position of the retinotopic visual output neurons.

### 2.1. Methods

**Figure 1:**
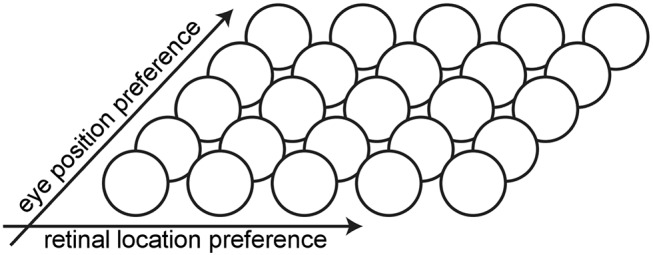
Architecture of the hardwired neural network model. The architecture of the hardwired neural network model consists of a single layer of visual output neurons. Each neuron is set to respond maximally to a unique combination of eye position and retinal location of the visual stimulus.

The architecture of the hardwired neural network model consists of a single layer of visual output neurons (Figure 1). The internal activation *h* of each visual neuron (Equation 1) consists of the sum of a component *h*_*r*_ encoding the retinal location *x* of the visual stimulus and a component *h*_*e*_ encoding the position *y* of the eye in the orbit. Each neuron is set to respond maximally to a unique combination of retinal location of the visual stimulus and eye position. The range of eye positions and retinal locations were defined within [−35, 35°] and [−10, 10°], respectively. The entire two dimensional space consisting of all possible combinations of eye position and retinal location of the visual stimulus is covered in integer steps of one degree in each dimension by the population of visual neurons. This resulted in a total of 71 × 21 = 1, 491 neurons.

(1) $$\begin{array}{r} h\left(x,y\right)=h_{r}\left(x\right)+h_{e}\left(y\right) \end{array}$$

The component of the internal activation *h*_*r*_ to each visual neuron that depends on the retinal location *x* of the visual stimulus is given by the Gaussian function

(2) $$\begin{array}{r} h_{r}\left(x\right)=h_{max_{r}}\times \exp\left(-\frac{{\left(x-\alpha \right)}^{2}}{2\sigma^{2}}\right) \end{array}$$

where *x* is the current retinal location of the visual stimulus, α is the neuron's preferred retinal location and σ is the width of the Gaussian tuning curve. The maximum value *h*_*ma*_*x*__*r*__ is achieved only when the visual stimulus is located at the neuron's preferred retinal location, i.e., when *x* = α. If the stimulus is located at any other retinal location, then the value of *h*_*r*_ corresponds to a Gaussian function of the difference between the stimulus location on the retina *x* and the neuron's preferred location α.

Similarly, the component of the internal activation *h*_*e*_ to each visual neuron that depends on the position *y* of the eye in the orbit is described by the Gaussian function

(3) $$\begin{array}{r} h_{e}\left(y\right)=h_{max_{e}}\times \exp\left(-\frac{{\left(y-\beta \right)}^{2}}{2\rho^{2}}\right) \end{array}$$

where *y* is the current position of the eye in the orbit, β is the neuron's preferred eye position and ρ is the width of the Gaussian tuning curve. The response of a given visual neuron is maximally amplified (i.e., *h*_*e*_(*y*) = *h*_*ma*_*x*__*e*__) when the eye shifts to the neuron's preferred eye position β. For all other eye positions the neuron's responses are modulated by a Gaussian function of the difference between the neuron's preferred eye position and the current position of the eye.

The instantaneous firing rate *v*_*i*_ of each visual neuron *i* in the hardwired model is given by

(4) $$\begin{array}{r} v_{i}=\frac{1}{1+\exp\left(-2\varphi \left(h-\theta \right)\right)} \end{array}$$

where the activation *h* is defined by Equation (1) and the sigmoid slope and threshold are denoted, respectively, by φ and θ.

### 2.2. Simulation Results

This simulation investigates the existence and nature of multiplicative gain modulation in retinotopic visual output neurons when the response profiles of both the retinotopic visual input component and eye position input component are modeled by Gaussian functions. The former modeling work of Salinas and Abbott (1996) used a linear function to model the influence of the position of the eye on visual responses. This is a particularly important issue because the responses of eye position neurons in the cortex have been reported to be a better fit to a Gaussian function (Marĉelja, 1980; Andersen et al., 1987; Dayan and Abbott, 2001; Wang et al., 2007). In the simulations presented here we show how multiplicative gain modulation of the visual neurons can occur even if a Gaussian function is used to model the term *h*_*e*_(*y*) denoting the component of the visual neuron activation that depends on eye position. The simulation parameters for the model are given in Table 1.

**Table 1:** Simulation parameters of hardwired model.

**Parameter**	**Symbol**	**Value**
Activation function slope	φ	1.9
Activation function threshold	θ	0.99
Population size	−	1, 491
Maximum eye position activation	*h*_*ma*_*x*__*e*__	0.485
Maximum retinal location activation	*h*_*ma*_*x*__*r*__	0.485
Width of eye position tuning curve	ρ	20°
Width of retinal location tuning curve	σ	6°

**Figure 2:**
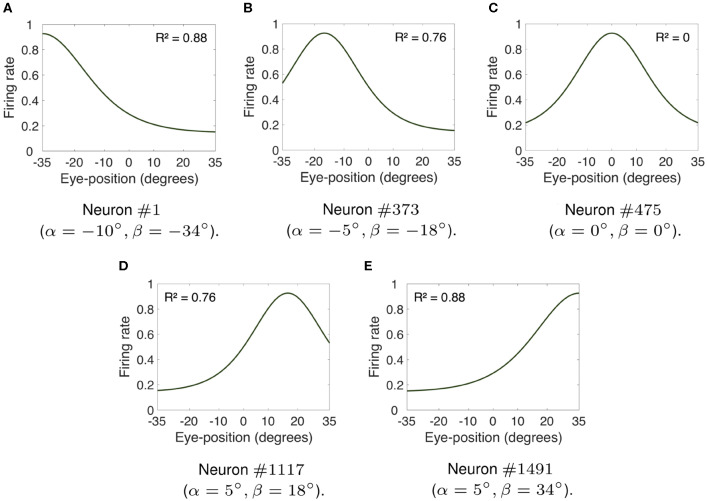
Firing rate responses of visual neurons in the hardwired model. **(A–E)** show, respectively, the responses of neurons #1, #373, #475, #1, 117, and #1, 491 when the visual target was kept at each neuron's preferred retinal location whilst the eyes shifted in integer steps of one degree to all positions within [−35, 35°]. The preferred retinal location of each of these neurons was set to −10, −5, 0, 5, and 10°. The preferred eye position of each neuron was −34, −18, 0, 18, and 34°, respectively. The coefficient of determination *R*^2^ provides a measure of linearity of the visual responses of each visual neuron. The values of *R*^2^ are defined within [0, 1], where 1 implies a perfect linear fit. In particular, whilst **(C)** shows that the response profile of neuron #475 was not linear (i.e., *R*^2^ = 0), all other subplots had more linear responses (i.e., values of *R*^2^ closer to 1). **(A,E)** had approximately linear monotonic response profiles covering the whole eye position space. Table 1 shows the simulation parameters for the model. These simulation results show that modeling the input components representing both the position of the eye and the retinal location of the visual stimulus using biologically realistic Gaussian functions results in a variety of forms of visual responses similar to parietal responses, including peaked and monotonic.

Figure 2 shows the visual responses of neurons in the hardwired model across different eye positions. For each subplot in Figure 2, the visual target was kept at the neuron's preferred retinal location whilst the eyes shifted in steps of one degree to all positions within [−35, 35°]. This range of eye positions has been observed as the functional limit on orbital position during natural visual exploration (Freedman and Sparks, 1997; Navarro et al., submitted). Previous modeling studies of brain function have also used this range of eye positions (Mender and Stringer, 2013, 2014; Navarro et al., 2018). Figure 2 shows the visual responses of neurons #1, #373, #475, #1, 117, and #1, 491. The preferred retinal location of the visual target for each of these neurons was respectively set to −10, −5, 0, 5, and 10°. The preferred eye position of each neuron was −34, −18, 0, 18, and 34°, respectively. The linearity of the visual responses shown in Figure 2 may be compared by calculating the *coefficient of determination* *R*^2^ of each response curve. The coefficient of determination is traditionally used in regression analysis to measure the success of predicting a dependent variable from independent variables (Rao, 1973). In other words, the coefficient of determination *R*^2^ provides statistical information about the *goodness-of-fit* of a model. In this work the coefficient of determination measures the goodness-of-fit of the visual responses across different eye positions to a *linear* model. The values of *R*^2^ are defined within [0, 1], where *R*^2^ equal to 1 indicates that a linear model perfectly predicts the visual responses. Such visual responses are referred to as *linear responses*. If *R*^2^ is equal to 0, then none of the visual responses can be predicted by a linear model. In this case, the visual responses are referred to as *non-linear responses*.

Figures 2A–E, respectively, show the responses of neurons #1, #373, #1, 117, and #1, 491. The response curves of neurons #1 and #1, 491 had a *R*^2^ value of 0.88, whilst neurons #373 and #1, 117 had response curves with *R*^2^ equal to 0.78. The values of *R*^2^ reflect the fact that the visual responses of neurons #1, #373, #1, 117, and #1, 491 were linear for the majority of eye positions. Figures 2A,E had approximately linear monotonic response profiles covering the whole eye position space. However, the peaked response curve of neuron #475 shown in Figure 2C had a *R*^2^ value equal to 0. Therefore, Figure 2 shows that modeling the influence of both eye position and retinal location of the visual stimulus using biologically realistic Gaussian functions results in a variety of functional forms of gain modulation of visual responses including both peaked and monotonic. This is a particularly relevant result because experimental studies have reported the existence of these different forms of visual responses in the cortex (Andersen and Mountcastle, 1983; Galletti and Battaglini, 1989; Lal and Friedlander, 1990; Brotchie et al., 1995; Galletti et al., 1995; Hoffmann, 1998; Trotter and Celebrini, 1999; Breveglieri et al., 2009; Merriam et al., 2013).

**Figure 3:**
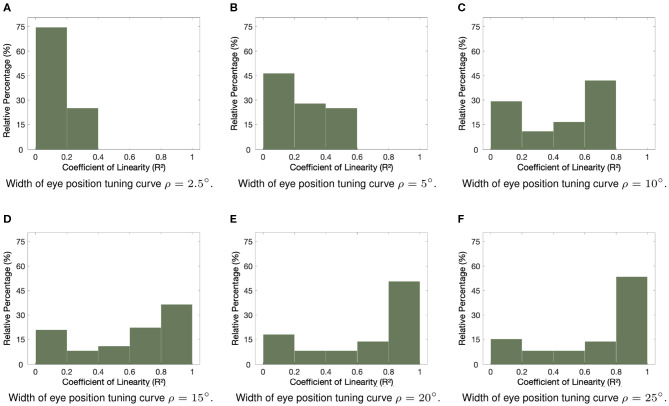
Influence of varying the width ρ of the eye position tuning curve in the hardwired model. The subplots show how the width ρ of the eye position tuning curve affected the linearity of the eye position gain modulation of the responses of the visual neurons in the hardwired model. Simulation parameters are given in Table 1. The coefficient of determination *R*^2^ was calculated to assess the linearity of the response curves of each neuron when the visual stimulus was in the neuron's preferred retinal location and the eye position varied in integer steps of one degree within [−35, 35°]. **(A–F)** show frequency histograms where individual visual neurons are binned according to their calculated value of *R*^2^. The plotted values are scaled to represent the relative percentages of the entire population of visual neurons. **(A–C)** show that the majority of responses were not linear (i.e., values of *R*^2^ were closer to 0) for values of ρ not greater than 10, whilst **(D–F)** show a higher proportion of more linear responses (i.e., values of *R*^2^ closer to 1) for greater values of ρ. These simulation results show a clear increase in the linearity of the visual responses caused by an increase in the width of the eye position tuning curve.

Next we study the impact of varying the width ρ of the eye position Gaussian tuning curve on the linearity of the eye position gain modulation of the responses of the entire visual population of neurons. We hypothesized that increasing the width ρ of the eye position tuning curve would increase the overall linearity of the gain modulation of visual responses by eye position. Figure 3 shows how the linearity of the eye position gain modulation of the responses of visual neurons changed for different values of ρ within [2.5, 25°]. The visual responses were tested for the same range of eye positions in Figure 2. The changes in the linearity of the responses were assessed using the coefficient of determination *R*^2^ for each response curve with the visual stimulus positioned in each neuron's preferred retinal location. Table 1 shows the simulation parameters for the model. The bars in each subplot represent the proportion of visual neurons in the hardwired model with responses resulting in values of coefficient of determination *R*^2^ from 0 to 1.0 with a bin size of 0.2. Figures 3A–C show that at least 57% of visual neurons had values of coefficient of determination *R*^2^ smaller than 0.5 when ρ was not greater than 10°. In other words, more than half of all visual responses were highly non-linear for small values of ρ. However, Figures 3D–F show that the values of coefficient of determination *R*^2^ of the majority of response curves are closer to 1.0 for greater values of ρ. In fact, Figures 3E,F show that more than 45% of all visual response curves in the hardwired model had *R*^2^ greater than 0.8. In particular, Figure 3 shows that the proportion of response curves with coefficient of determination *R*^2^ not greater than 0.2, decreased from approximately 75 to 17% when the value of ρ increased from 2.5 to 25°. These simulation results show that a higher proportion of linear responses is obtained with greater values of ρ.

**Figure 4:**
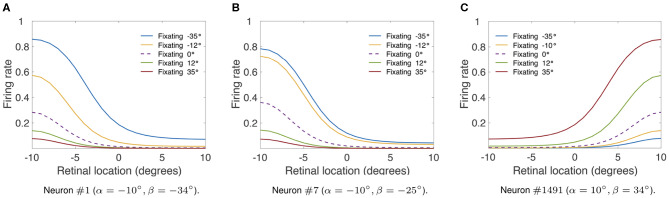
Modulation of visual neuron responses by eye position in the hardwired model. The figure shows the gain modulation by eye position of visual responses of neurons in the hardwired model. **(A)** Shows the visual responses of neuron #1 (α = −10°, β = −34°), **(B)** shows the visual responses of neuron #7 (α = −10°, β = −25°) and **(C)** shows the visual responses of neuron #1491 (α = 10°, β = 34°). The x-axis (abscissa) indicates the retinal location of the visual stimulus. Table 1 gives the simulation parameters for the model. Each curve corresponds to fixations with the following eye positions: −35°, −12°, 0°, 12° and 35°. The broken line represents fixation straight ahead (i.e., 0°). The symbols were obtained by multiplying the straight-ahead fixation response curve by a different constant value for each of the other response curves. The response curves in each subplot show that there is a form of modulation of the visual responses by eye position that is approximately multiplicative over the simulated retinal locations of the stimulus with a sigmoidal transfer function (Equation 7).

Finally, we investigate whether the linear responses in the hardwired model (Figure 2) would produce multiplicative gain modulation by eye position over all retinal locations of the stimulus. Table 1 shows the simulation parameters for the model. In particular ρ was set equal to 15°. Figure 4 shows the responses of neuron #1, neuron #7, and neuron #1, 491 in the hardwired model as a function of the retinal location of the visual stimulus and eye position. For each different fixation made to −35, −12, 0, 12, and 35° the responses of each neuron were recorded for all retinal stimulus locations within [−10, 10°] in steps of one degree. To demonstrate the multiplicative nature of the eye position gain modulation of the neuronal responses, symbols were plotted in Figure 4 by multiplying the response corresponding to the straight ahead fixation (broken line) by a constant value for each fixation. The precise alignment of the symbols over the response curves shows that the visual responses are modulated multiplicatively by the position of the eye. Thus, Figure 4 shows that the form of gain modulation by eye position of the visual responses of neuron #1, neuron #7, and neuron #1491 is multiplicative.

In this section, we presented a hardwired model of multiplicative eye position gain modulation of the responses of visual neurons in the cortex. In Figure 2, we showed that modeling both the retinal preference and the eye position preference of visual neurons using a biologically realistic Gaussian function resulted in a variety of forms of eye position gain modulation across the entire population of visual output neurons. This is a particularly important result as different forms of eye position gain modulation of visual neurons have been observed in the cortex (Galletti and Battaglini, 1989; Lal and Friedlander, 1990; Galletti et al., 1995; Hoffmann, 1998; Trotter and Celebrini, 1999; Breveglieri et al., 2009; Merriam et al., 2013). Next we showed that varying the width of eye position tuning curves increased the linearity of eye position gain modulation across the entire population of visual neurons (Figure 3). In Figure 4, we showed that the model can produce visual neurons with multiplicative gain modulation by eye position over all retinal locations of the stimulus.

In the next section we study a self-organizing model of how multiplicative eye position gain modulation may develop in the brain.

## 3. Self-Organizing Model

In this section we present simulation results of a self-organizing neural network model of the development of multiplicative gain modulation of visual responses by eye position. That is, we show how the synaptic connectivity could develop through a biologically plausible learning process. In the self-organizing model, the visual output neurons are driven by two explicitly modeled populations of input neurons. One input population encodes the retinotopic position of the stimulus, whilst the other input population encodes the position of the eyes. In a manner analogous to the hardwired model, the response profiles of both populations of input neurons are modeled using biologically realistic Gaussian functions. The activations of the visual output neurons were driven by additively combining the inputs from these two populations of input neurons as would occur in the brain. A sigmoid transfer function was used to model the firing rate responses of the visual output neurons in the self-organizing model. In this section we investigate the influence of the width of the eye position input neuron tuning curves on the self-organization of linear eye position gain modulation at preferred retinal stimulus locations and the development of multiplicative eye position gain modulation across all retinal stimulus locations. During the unsupervised self-organizing learning process described in this section output neurons compete to respond to specific input patterns. This means that output neurons learn to differentiate responses properties for each input pattern present to the model during training. In this manuscript each input pattern consists of different randomized combinations of eye position and retinal stimulus location that are applied to the input layer of the model. The output layer does not need to be topologically organized.

### 3.1. Methods

**Figure 5:**
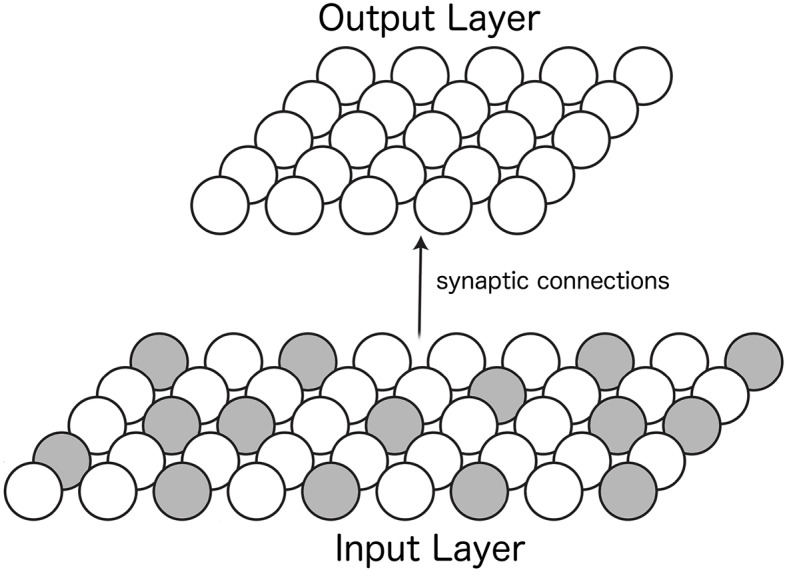
Architecture of the self-organizing neural network model. Afferent synaptic connections from neurons in the input layer **(Bottom)** are projected to neurons in the output layer **(Top)**. The responses of neurons within the input layer depend on either the position of the eye or the retinal location of the visual stimulus. The two colors of neurons in the input layer represent these two different forms of neuronal input responses. A Hebbian learning rule is used to modify the strengths of the feedforward synaptic connections from the input layer to the output layer during training.

The architecture of the self-organizing neural network model consists of two layers of neurons. The first layer, the *input layer*, had a mixture of neurons with responses depending on either the position of the eye or the retinal location of the visual stimulus. Feedforward synaptic connections from neurons in the input layer to neurons in the second layer, the *output layer*, were updated with a Hebbian learning rule during training. After training, neurons in the output layer were expected to develop the form of retinotopic visual responses with multiplicative eye position gain modulation shown in the previous section (Figures 2, 4). Figure 5 shows the architecture of the model.

Neurons in the input population had responses depending on either the position of the eye or the retinal location of the visual stimulus. Neurons with responses depending on the position of the eye had a Gaussian internal activation given by Equation (3). Each of these neurons is set to fire maximally to a unique eye position within [−35, 35°] in integer steps of one degree. Neurons with responses depending on the retinal location of the visual stimulus had a Gaussian internal activation described by Equation (2), where the range of preferred retinal locations is defined within [−10°, 10°] in integer steps of one degree. This resulted in a total of 71+21 = 92 input neurons. The instantaneous firing rate *v*_*j*_ of each input neuron *j* is given by the sigmoid activation function

(5) $$\begin{array}{r} v_{j}=\frac{1}{1+\exp\left(-2\varphi \left(h-\theta \right)\right)} \end{array}$$

where the sigmoid slope and threshold are denoted by φ and θ, respectively, and the activation *h* is defined by either Equation (2) if the neuron had responses depending on the retinal location of the visual stimulus, or Equation (3) if the neuron had responses depending on the position of the eye.

The output population consists of a total of 100 neurons. The internal activation *h*_*i*_ of each neuron *i* in the output layer is governed by

(6) $$\begin{array}{r} \tau_{h}\frac{dh_{i}}{dt}=-h_{i}+\underset{j}{\sum }w_{ij}v_{j} \end{array}$$

where *w*_*ij*_ is the strength of the synapse from input neuron *j* to output neuron *i*. The parameter τ_*h*_ is a time constant common for all output neurons.

The instantaneous firing rate *v*_*i*_ of each output neuron *i* is given by the sigmoid transfer function

(7) $$\begin{array}{r} v_{i}\left(t\right)=\frac{1}{1+\exp\left(-2\varphi \left(h_{i}\left(t\right)-p_{\pi }-\theta \right)\right)} \end{array}$$

where *h*_*i*_ corresponds to activation of output neuron *i* and φ and θ, respectively, denote the sigmoid slope and threshold. The parameter *p*_π_ controls the level of competition amongst output neurons by limiting the proportion of output neurons that are active at any time. For instance, *p*_π_ is set to the top fifteenth percentile activation value within the output population when the sparseness percentile π is set to 85. This form competition within the output layer has been previously used in neural network models of the primate visual system (Rolls, 2012). In the cortex this form of lateral inhibition is implemented via inhibitory interneurons (Dayan and Abbott, 2001).

Each output neuron receives synaptic connections from a unique randomly assigned subset of input neurons corresponding to ϕ percent of the input population. Before training, the weights of these feedforward synaptic connections are initialized to random values within [0, 1]. During training the synaptic weights are updated by a Hebbian learning rule given by

(8) $$\begin{array}{r} \frac{dw_{ij}}{dt}=\varrho v_{i}v_{j} \end{array}$$

where ϱ is the learning rate, *v*_*j*_ is the current firing rate of the presynaptic neuron *j* (Equation 5) and *v*_*i*_ is the current firing rate of the postsynaptic neuron *i* (Equation 7).

To prevent unbounded growth of the synaptic weights during training (Dayan and Abbott, 2001), after each weight update the length of the synaptic weight vector **w**_*i*_ for each output neuron *i* is renormalized by setting

(9) $$\begin{array}{r} {\text{w}}_{i}: =\frac{{\text{w}}_{i}}{∥{\text{w}}_{i}∥} \end{array}$$

The experimental work of Royer and Paré (2003) provides evidence for renormalization of synaptic weights in the brain.

The self-organizing model makes no assumptions about the temporal dynamics of eye movements relative to the changing positions of visual stimuli within the environment. Therefore, training involves applying lots of different randomized combinations of eye position and retinal stimulus location to the input layer of the model. The activity is propagated from the input layer to the visual output layer, where a small portion of output neurons win the competition to respond to the current inputs. Next, the feedforward synaptic weights from the active input neurons to the active output neurons are strengthened by Hebbian learning. This training process eventually endows the visual output neurons with their learned response properties.

Training lasts for 10 epochs. In each training epoch, the model is exposed to time-varying combinations of eye position and stimulus retinal location. Specifically, each epoch consists of periods in which a visual stimulus is presented for 2 s at each retinal location within [−10, 10°] in integer steps of one degree. During each of these periods the model performs 14 saccades at a constant velocity of 400°/*s* to a different random eye position within [−35, 35°]. These saccades are interleaved with 15 fixations, each lasting for 300 ms. Therefore, training was completed after approximately 420*s* of simulated time. The differential Equations (6) and (8) were numerically integrated using a Forward-Euler scheme with numerical time step set as one-tenth of the time constant τ_*h*_.

### 3.2. Simulation Results

This simulation investigates the development of visual output neurons with multiplicative gain modulation by eye position when the input population consists of a mixture of neurons with Gaussian responses depending on either the position of the eye or the retinal location of the visual stimulus. The influential model by Salinas and Abbott (1996) used a linear function to model how visual responses depended on the position of the eyes. However, the response profiles of eye position neurons in parietal cortex have been observed to be a better fit to a Gaussian function than to a linear function (Marĉelja, 1980; Dayan and Abbott, 2001). Furthermore, the efferent synaptic connections were both excitatory and inhibitory for the same neuron in the model of Salinas and Abbott (1996), which violates the Dale's law (O'Donohue et al., 1985). The synaptic connections within the self-organizing model discussed in this section do not violate the Dale's law. Table 2 gives the parameters of the self-organizing model.

**Table 2:** Simulation parameters of self-organizing model.

**Parameter**	**Symbol**	**Value**
Activation function slope	φ	4.5
Activation function threshold	θ	0
Activation time constant	τ_*h*_	100*ms*
Input neuron population size	–	92
Learning rate	ϱ	0.05
Maximum eye position activation	*h*_*ma*_*x*__*e*__	2.0
Maximum retinal location activation	*h*_*ma*_*x*__*r*__	2.0
Number of training epochs	–	10
Output neuron population size	–	100
Sparseness percentile	π	90%
Synaptic connectivity	ϕ	10%
Width of eye position tuning curve	ρ	15^°^^†^
Width of retinal tuning curve	σ	6°

† *The default value of ρ was 15°. However, this value was altered in some of the simulations*.

**Figure 6:**
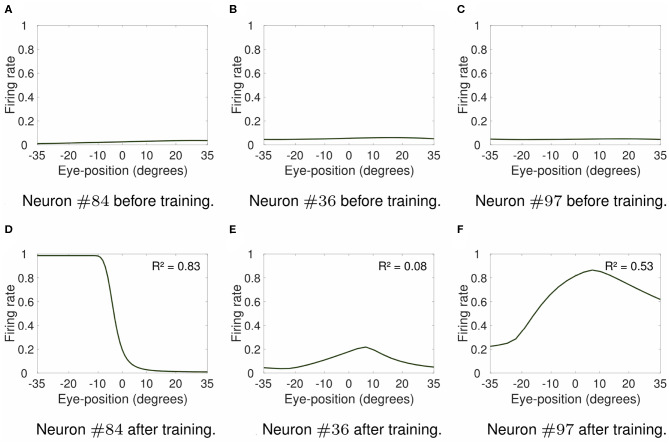
Firing rate responses of output visual neurons in the self-organizing model. Each subplot shows the firing rate responses of output neurons #84, #36, and #97 in the self-organizing model either before training (top row) or after training (bottom row). The responses are plotted for the preferred retinal locations of neurons #84, #36, and #97 at −10, 0, and 2°, respectively. These neurons were chosen to show the variety of different forms of visual output responses in the trained model. Table 2 shows the simulation parameters for the model. The value of ρ was set to 15. The comparison of responses prior to training **(A–C)** to the respective responses after training **(D–F)** shows that training changed the responses of these output neurons. Particularly, the response curves of neurons #84, #36, and #97 in the trained model (bottom row) resemble the response curves shown in Figure 2 of neurons #1 (Figure 2A), #475 (Figure 2C), and #1117 (Figure 2D) in the hardwired model, respectively. The values of the coefficient of determination *R*^2^ for each response curve in the trained model show that the form of visual responses in the output layer after training varied from non-linear (e.g., *R*^2^ = 0.08 for neuron #36) to more linear responses (e.g., *R*^2^ = 0.53 and *R*^2^ = 0.83 for neurons #97 and #84, respectively). These simulation results show that the use of more biologically realistic Gaussian functions to model the response profiles of input neurons encoding either eye position or retinal location of the visual stimulus enables the self-organizing model to develop a variety of forms of visual responses, including peaked and monotonic, similar to cortical responses.

Figure 6 shows the visual responses across different eye positions of selected output neurons before training (top row) and after training (bottom row). Figures 6A,D show the responses of neuron #84 when the visual stimulus was presented at retinal location −10°. For the responses of neuron #36 shown in Figures 6B,E the visual stimulus was presented at retinal location 0°. Finally, the visual stimulus was presented at retinal location 2° for responses of neuron #97 shown in Figures 6C,F. The comparison of the responses in the untrained model (Figures 6A–C) with the respective responses in the trained model (Figures 6D–F) shows how training changed the responses of output neurons. For instance, the response curve of output neuron #84 after training resembles the response curve of neuron #1 shown in Figure 2, with similar coefficients of determination *R*^2^ of 0.83 and 0.88, respectively. Likewise, the response curves of neurons #36 (Figure 6E) and #97 (Figure 6F) in the trained model resembles the responses of neurons #475 (Figure 2C) and #1117 (Figure 2D) in the hardwired model. The values of coefficient of determination *R*^2^ of output neuron #97 in the trained model and neuron #1117 in the hardwired model were 0.53 and 0.78, respectively. The values of coefficient of determination *R*^2^ were very similar for both output neuron #36 in the self-organizing model after training (*R*^2^ = 0.08) and neuron #475 in the hardwired model (*R*^2^ = 0). Therefore Figure 6, together with the analysis of the coefficient of determination *R*^2^ of the visual response curves, show that modeling the response profiles of eye position and retinotopic input neurons with more biologically realistic Gaussian functions drives the self-organization of a variety of visual output responses similar to the responses in the hardwired model (Figure 2).

**Figure 7:**
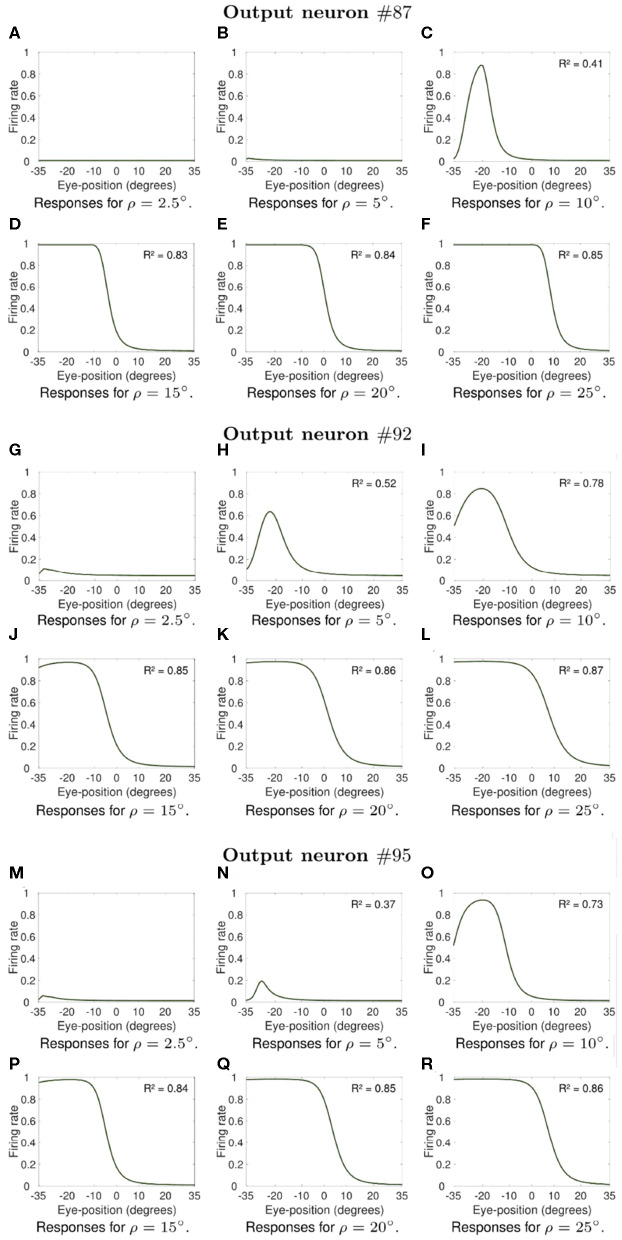
Influence of varying the width ρ of the eye position tuning curve in the self-organizing model. The subplots show how the width ρ of the eye position tuning curve affected the self-organization of the visual responses of neuron #87, neuron #92 and neuron #95. Each subplot shows the responses of these output neurons in the trained model when the visual stimulus was presented at retinal location −10° and the eyes shifted from [−35°, 35°] in integer steps of one degree. Table 2 gives the simulation parameters for the model. Each subplot indicates the value of ρ that was used during training and testing. For each of these neurons, the subplots in the top row show how increasing the value of ρ changes the firing rate responses from showing little activity (**A,B** for neuron #87, **G** for neuron #92, and **M** for neuron #95) to presenting an approximately Gaussian response profile (**C** for neuron #87, **H,I** for neuron #92, and **N,O** for neuron #95). Plots in the bottom row for each of these output neurons (**D–F**,**J–L**,**P–R**) show that training the model with greater values of ρ resulted in more sigmoidal response profiles. These simulations results show that the width of the eye position tuning curve has a large impact on the self-organization of the functional form of the output responses, with the profiles passing from peaked to monotonic for the majority of output neurons as ρ increases from 10° to 25°. The values of the coefficient of determination *R*^2^ in each subplot show how the width ρ of the tuning curve of the eye position neurons affected the degree of linearity of the self-organized output responses. The values of *R*^2^ were only calculated when the output neuron had significant activity for a range of eye positions after training.

**Figure 8:**
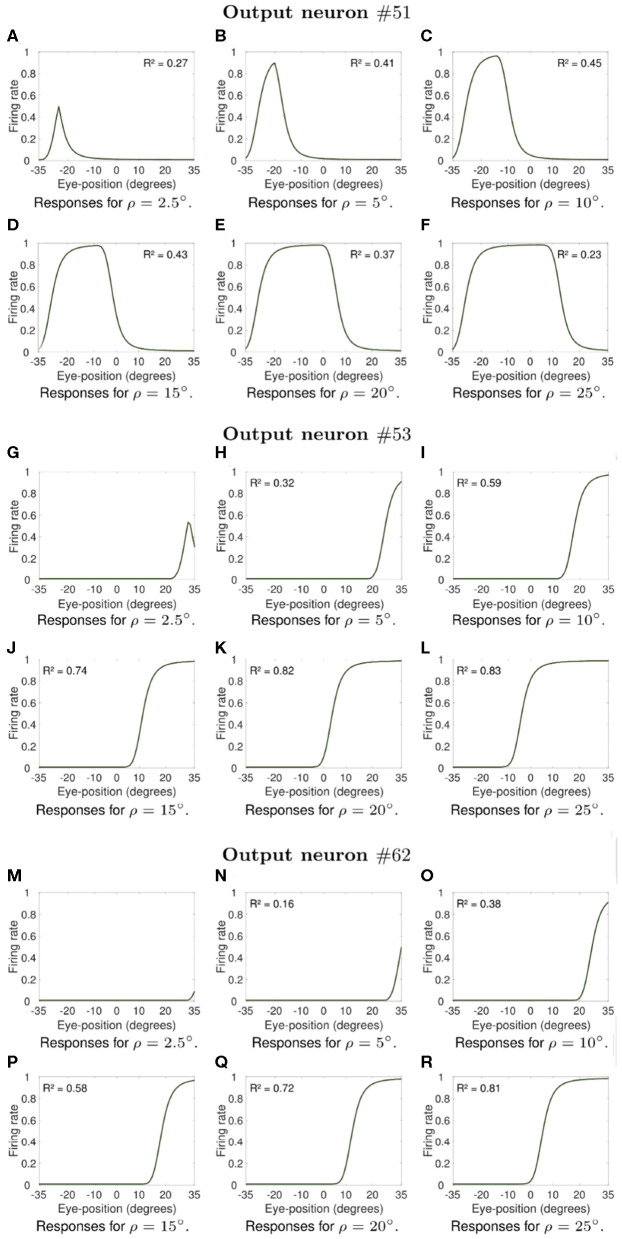
Influence of varying the width ρ of the eye position tuning curve in the self-organizing model. The subplots shows extra examples of how the width ρ of the eye position tuning curve affected the self-organization of the visual responses of output neurons #51, neuron #53, and neuron #62. Similar to Figure 7, the visual stimulus was presented at retinal location −10° and the eyes shifted from [−35°, 35°] in integer steps of one degree. Table 2 gives the simulation parameters for the model. The value of ρ for training and testing is indicated for each subplot. These subplots show that an increase in the value of ρ changes the firing rate responses from showing little activity **(A,G,M,N)** to presenting an approximately Gaussian response profile **(B–F)**. The remaining subplots **(H–L,O–R)** show that training the model with greater values of ρ resulted in more sigmoidal response profiles. These simulations results show that the width of the eye position tuning curve has a large impact on the self-organization of the functional form of the output responses, with the profiles passing from peaked to monotonic as ρ increases from 10° to 25° for the majority of output neurons. The responses of neuron #51, however, were not monotonic even for greater values of ρ. The values of the coefficient of determination *R*^2^ in each subplot show how the width ρ of the tuning curve of the eye position neurons affected the degree of linearity of the self-organized output responses. The values of *R*^2^ were only calculated when the output neuron had significant activity for a range of eye positions after training.

Next we investigate what would be the impact of varying the width ρ of the eye position tuning curve on the self-organization of responses in the output population of visual neurons. In particular, we investigate whether greater values of ρ would result in an increase in the linearity of how visual output responses vary with eye position in the self-organizing model, similar to the effects presented in previous section. The model was individually trained as described above for the following different values of ρ: 2.5, 5, 10, 15, 20, and 25°. Simulation parameters are given in Table 2. Figures 7, 8 show the influence of varying ρ on the self-organization of linear responses for neurons #87, #92, #95, #51, #53, and #62. The responses of each of these output neurons in the trained model were recorded when the visual stimulus was presented at retinal location −10° and the eyes shifted from [−35, 35°] in integer steps of one degree. Figures 7, 8 show that increasing the value of ρ drives the development of more sigmoidal output visual responses for the majority of output neurons within the parameter space that was tested. These simulation results show that the width ρ of the tuning curve of the eye position neurons has an impact on the self-organization of the degree of linearity of the output responses, as indicated by the values of the coefficient of determination *R*^2^ in each subplot. The values of *R*^2^ were only calculated when the output neuron had significant activity for a range of eye positions. Figures 7A,B show that for values of ρ not greater than 5° the firing rate of output neuron #87 in the trained model was close to zero for all eye positions. Figure 7C shows a more Gaussian response profile when ρ = 10° for the same output neuron. Figures 7D–F, however, show that for greater values of ρ the response profile of output neuron #87 after training is more sigmoidal across all eye positions. Figures 7G,M show that output neuron #92 and output neuron #95, respectively, were mostly unresponsive for ρ = 2.5°. However, the response profiles of these neurons were approximately Gaussian for ρ = 5° and ρ = 10° (Figures 7H,I for output neuron #92, and Figures 7N,O for output neuron #95). Figures 7J–L,P–R show that the response profiles of output neurons #92 and #95 in the trained model were approximately sigmoidal for all eye positions within [−35, 35°] when the values of ρ were greater than 10°. Figures 8A,G,M,N show that for values of ρ not greater than 5° the firing rate of output neurons #51, #53, and #62 in the trained model was close to zero for all tested eye positions. Figures 8I–L,O–R shows that for greater values of ρ the response profiles of output neurons #53 and were approximately sigmoidal.

Moreover, the coefficient of determination *R*^2^ increases from values not greater than 0.52 for smaller values of ρ (Figures 7C,H, 8H–I,N–P) to values greater than 0.7 for larger values of ρ (Figures 7D–F,I–L,O–R, 8J–L,Q–R). The responses of neuron #51 (Figures 8A–F) show that the for some output neurons the responses were not sigmoidal after training even with greater values of ρ. The effects of varying ρ presented in Figures 7, 8 were generally observed amongst a large population of output visual neurons after training.

**Figure 9:**
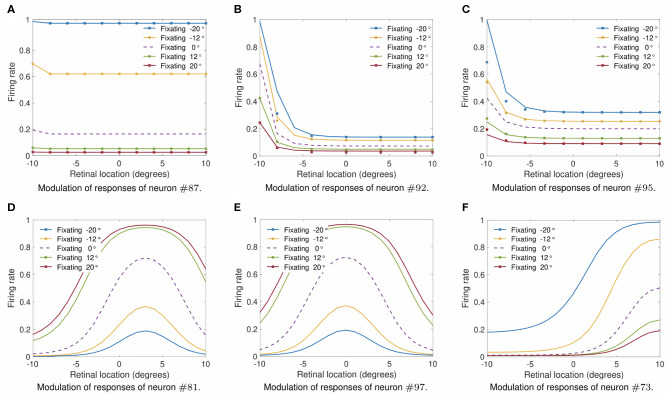
Modulation of visual neuron responses by eye position in the self-organizing model after training. The figure shows the gain modulation by eye position of the visual responses of neurons #87 **(A)**, #92 **(B)**, #95 **(C)**, #81 **(D)**, #97 **(E)**, and #73 **(F)** after training in the self-organizing model. The x-axis (abscissa) indicates the retinal location of the visual stimulus. The simulation parameters for the model are shown in Table 2. The value of ρ was set to 17°. Each curve corresponds to fixations with the following eye positions: −20°, −12°, 0°, 12°, and 20°. The broken line represents fixation straight ahead. The symbols in subplots **(A–C)** were obtained by multiplying the straight-ahead fixation response curve by a different constant value for each of the other response curves. The general alignment of the symbols over the response curves shows that the form of modulation of the visual responses by eye position is approximately multiplicative over all the retinal location of the stimulus within the parameter space that was tested. Each subplot shows that training was capable of driving the development of output responses with a form of eye-position gain modulation that is approximately multiplicative.

Finally, we studied whether output neurons displayed multiplicative gain modulation by eye position over all retinal locations of the stimulus. Figure 9 shows how the output responses of neuron #87, neuron #92, neuron #95, neuron #81, neuron #97, and neuron #73 changed for different fixations when the visual target was shifted across the retina to locations within [−10, 10°] in integer steps of one degree. Table 2 gives the simulation parameters for the model. The value of ρ was set to 17°. The responses of each of these output neurons were tested for fixations to −20, −12, 0, 12, and 20°. To demonstrate the multiplicative nature of the eye position gain modulation of the neuronal responses, symbols were plotted in Figures 9A–C by multiplying the response corresponding to the straight ahead fixation (broken line) by a constant value for each fixation. The alignment of the symbols over the response curves shows that the visual responses are approximately modulated in a multiplicative manner by the position of the eye. This is the same methodology used in the influential work of Salinas and Abbott (1996). Figures 9D–F shows the form of modulation by eye-position of the responses neurons #81, #97, and #73. Therefore the simulation results in Figure 9 show that training had the effect of developing a form of gain modulation by eye position over all retinal locations of the stimulus that is approximately multiplicative for output neurons #87, #92, #95, #81, #97, and #73.

## 4. Discussion

In this work, we study both hardwired and self-organized neural network models of how multiplicative gain modulation of visual responses by the position of the eye may develop in the brain. A variety of forms of visual responses, such as peaked, sigmoidal, and more linear responses, have been observed in cortical areas (Andersen and Mountcastle, 1983; Galletti and Battaglini, 1989; Lal and Friedlander, 1990; Brotchie et al., 1995; Galletti et al., 1995; Hoffmann, 1998; Trotter and Celebrini, 1999; Breveglieri et al., 2009; Merriam et al., 2013). Previous modeling studies (Salinas and Abbott, 1996) used a linear function to model the dependence of the visual responses on eye position. However, the response profiles of eye position neurons in parietal areas have been reported to be a better fit to Gaussian than to linear functions (Marĉelja, 1980; Andersen et al., 1987; Dayan and Abbott, 2001; Wang et al., 2007). The simulation results presented in this work showed how multiplicative gain modulation by eye position may develop using inputs from eye position neurons with Gaussian response profiles. We started by studying a hardwired model of how cortical visual neuron responses depend on both the retinal location of the visual target and the position of the eyes. The simulation results in Figure 2 showed a variety of different functional forms of gain modulation by eye position of visual responses in the hardwired model that were similar to cortical responses. In particular, the responses in Figures 2A,E in the hardwired model were approximately linear for all eye positions when the visual stimulus was at the neuron's preferred retinal location. We also investigated how varying the width ρ of the eye position Gaussian tuning curve affected the functional form, including the linearity, of the eye position gain modulation of visual responses. We showed in Figure 3 that an increase in the width of the eye position tuning curve resulted in more linear responses. These simulation results showed that more biologically realistic Gaussian tuning curves, modeling how visual responses depend on the retinal location of the visual stimulus and on the position of the eye, resulted in different functional forms of gain modulation by eye position similar to those seen in parietal cortex, including responses that were linear across different eye positions. In Figure 4, we showed that such visual responses represent a form of gain modulation that is multiplicative by eye position.

A further important contribution of our work is the investigation of how such a variety of different functional forms of gain modulation by eye position, as well as multiplicatively gain modulated visual responses, would self-organize in a neural network model of cortical neurons. Our self-organizing model consisted of a population of input neurons sending feedforward synaptic connections to an output population of visual neurons. The input population consisted of a mixture of neurons with Gaussian responses depending on either the position of the eye or the retinal location of the visual stimulus. Training consisted of presenting the network with lots of different randomized combinations of retinal stimulus location and eye position. The weights of the synaptic connections were updated during training using a Hebbian learning rule (Equation 8). Figure 6 showed that output neurons in the trained model had responses similar to the ones plotted for the hardwired model in Figure 2. These simulation results showed that the model successfully self-organized the forms of visual responses observed in the parietal cortex including peaked and monotonic. In particular, Figure 6D showed that the responses of neuron #87 in the trained model were monotonic and somewhat similar to the approximately linear responses of neuron #1 in the hardwired model (Figure 2A). The simulation results in Figure 9 showed that training had the effect of producing output neurons with a form of eye-position gain modulation that is approximately multiplicative. Additionally, in Figure 7 we showed how varying the width ρ of the tuning curve of eye position input neurons affected the self-organization of output neuron responses. Similar to the effects presented in Figure 4, the simulation results in Figure 7 showed that increasing the width of the eye position tuning curve resulted in the self-organization of responses that varied more monotonically with eye position. However, the neuronal response profiles shown in Figures 7D–F,J–L,P–R, 8I–L,P–R were only linear over a narrow range of eye positions. This may have been due to the relatively high value of the slope ϕ of the sigmoid transfer function (Equation 7) used in the simulations. A larger slope ϕ produces a more sharp increase in the neuronal firing rate *v*_*i*_ as the input activation *h* varies. Additional simulations with the hardwired model varying the sigmoid slope φ between 1 and 10 showed a change in the response profile of neurons from flat responses across all eye-positions (φ smaller than 5) to response profiles with much larger width of the Gaussian profile (φ greater than 5). In terms of self-organization, although varying the value of φ in isolation degraded the performance of the self-organization process, the model was still capable of developing the form of responses previously discussed. A similar effect was observed when varying in isolation the parameter *p*_π_ of the self-organizing model. In summary, changing these parameters in isolation made the self-organization behavior less robust, and the exploration over the full parameter set is beyond the scope of this paper.

Gain modulated responses in parietal cortex are critical for coordinate transformation from eye-centered or retinocentric frames of reference to other body-centered frames of reference more suitable to guide motor behaviors, such as head-centered and hand-centered that have been observed in cortex. Here we have shown the emergence of different functional forms of gain modulation, including peaked and monotonic modulation, in both a hardwired and a self-organizing neural network model of cortical visual neurons.

## Data Availability Statement

All datasets generated for this study are either included in the article/**Supplementary Material**, or can be obtained upon request.

## Author Contributions

DN, HS, and SS designed the research, reviewed, and edited the paper. DN performed the research, data analysis, validation, visualization, and wrote first draft of the paper.

## Conflict of Interest

The authors declare that the research was conducted in the absence of any commercial or financial relationships that could be construed as a potential conflict of interest.
